# Effects of organic fertilizer replacing chemical fertilizer on organic carbon mineralization and active carbon fractions in yellow paddy soil of Guizhou Province

**DOI:** 10.1371/journal.pone.0323801

**Published:** 2025-06-02

**Authors:** Jie Wei, Sanwei Yang, Xiaoli Wang, Jianjun Duan, Ting ting Mei, Mingrui Li, Shengmei Yang, Fangchi Wang

**Affiliations:** 1 College of Agriculture, Agricultural College of Guizhou University, Guizhou University Tobacco College, Guizhou University, Guiyang, China; 2 Key Laboratory of Tobacco Quality Research of Guizhou Province, College of Tobacco, Guizhou University, Guiyang, China; University of Minnesota, UNITED STATES OF AMERICA

## Abstract

The aim was to decrease chemical fertilizer use and improve soil carbon sequestration. Replacing 50% chemical nitrogen fertilizer with organic fertilizer can inhibit the mineralization of organic carbon in yellow paddy soil by increasing the active organic carbon components. Four fertilization treatments (no fertilization, conventional fertilization, 50% organic fertilization and 50% chemical nitrogen fertilization, and organic fertilization instead of chemical nitrogen addition) were used to investigate the effects of using organic fertilizer instead of chemical fertilizer on soil organic carbon mineralization and active organic carbon components in paddy fields. The soil organic carbon, total nitrogen, available phosphorus, and available potassium contents were markedly higher for the organic fertilizer treatment than the no fertilization treatment. Compared with the application of chemical fertilizer alone, the substitution of chemical fertilizer with organic fertilizer significantly increased soil pH and significantly decreased the content of available potassium. The cumulative soil organic carbon mineralization rates for all treatments decreased during the incubation period. The ROC, dissolved organic carbon, and MBC contents were in 24.46%, 55.45%, and 17.60% higher, respectively, before and 19.34%, 74.98%, and 66.83%, respectively, after mineralization for 50% organic fertilization than no fertilization. Compared with the single application of chemical fertilizer, the ROC and DOC in the 1/2NPKM treatment increased significantly by 10.32% and 56.03% respectively after mineralization (p < 0.05), while the MBC in the M treatment decreased significantly by 12.05% before and 27.05% after mineralization (p < 0.05). The decrease in ROC was the most significant. Soil organic carbon mineralization was negatively correlated with SOC and active carbon fractions, and SOC was positively correlated with active carbon fractions. In summary, replacing 50% of chemical fertilizer with organic fertilizer inhibited soil organic carbon mineralization, which would improve carbon sequestration and fertilization. ROC and MBC were the main organic carbon sources mineralized.

## Introduction

Guizhou yellow soil has the characteristics of strong acidity, rich organic matter, strong water and fertilizer retention ability, deep soil layer and good permeability [1]. The pH value is usually between 4.5-5.5, which has strong acidity, but also has good water and fertilizer retention ability [2]. It is mainly distributed in the Guizhou Plateau between 23.5°and 30° north and south latitudes, and is one of the most important yellow soil distribution areas in China [3].

Long-term excessive application of chemical fertilizers readily causes soil acidification, hardening, and other problems [4]. Organic fertilizers contain various nutrients required by crops, and fertilizers have stable and lasting effects. Organic fertilizers are also rich in organic matter and can improve soil [5]. In recent years, the Chinese state has vigorously promoted the replacement of chemical fertilizers with organic fertilizers, and it is proposed in the “Action Plan for Chemical Fertilizer Reduction by 2025” to further increase the area in southwest China in which organic fertilizers replace chemical fertilizers [6]. Increasing the soil organic carbon (SOC) content is vital for improving soil quality, as SOC is a key indicator of soil fertility [7]. SOC mineralization is the main pathway through which the soil carbon pool releases CO_2_ into the atmosphere. A change in the SOC content of 0.1% will change the CO_2_ concentration in the atmosphere by 1 mg/L [8]. Active SOC means organic carbon that is strongly active with a fast turnover rate and that is readily decomposed. The main types of active SOC are readily oxidizable organic carbon (ROC), dissolved organic carbon (DOC), and microbial biomass carbon (MBC) [9,10], which are more sensitive than other types of SOC to changes in the soil carbon pool [11]. The effects of replacing chemical fertilizers with organic fertilizers on SOC mineralization in, and active carbon component contents of farmland soil should be studied to improve our understanding of how we can decrease chemical fertilizer use, improve the carbon sequestration capacity of soil, and alleviate climate warming [12].

The effects of replacing chemical fertilizers with organic fertilizers on SOC mineralization and active carbon components have been studied in China and other countries. Organic fertilizers mainly affect organic carbon mineralization by improving the structure and increasing the organic matter content of soil and by increasing microbial community richness [13]. The cumulative mineralization rate and cumulative mineralization of SOC in paddy soil were lower when organic fertilizer was used to replace 25% and 50% of chemical fertilizer and when organic fertilizer was added compared with no fertilization, meaning that adding organic fertilizer increased the amount of carbon sequestered in the paddy soil [14–16]. Replacing 15% of the applied chemical fertilizer with organic fertilizer at a single application can reduce CO_2_ emissions from farmland [17]. Replacing 75% of the applied chemical fertilizer with bio-organic fertilizer can markedly increase the SOC content of citrus orchard soil and decrease the mineralization and cumulative mineralization rates [18]. Most studies have found that replacing 50% of the applied chemical fertilizer with organic fertilizer markedly increases the DOC, MBC, and ROC contents [19–21]. Replacing 24% of the applied chemical fertilizer with organic fertilizer at a single application markedly increases the DOC, MBC, ROC, and SOC contents [22]. Compared with single application of organic fertilizer, organic fertilizer replacing 20% of chemical fertilizer reduced ROC content and MBC content [23].The differences between the results of the studies mentioned above may have been caused by differences in many factors, such as the climate, organic fertilizer type, soil fertility, soil parent material, soil type, and tillage mode.

The effects of using organic fertilizer instead of chemical fertilizer on organic carbon mineralization and active carbon components in yellow paddy soil in Guizhou are still unclear. In this study, the effects of replacing different proportions of chemical fertilizer with organic fertilizer on SOC mineralization and active carbon components were investigated by performing field experiments and indoor mineralization cultures using yellow paddy field soil in Guizhou. The results were expected to improve our theoretical understanding of carbon fixation and fertilization in yellow paddy field soil in Guizhou.

## Materials and methods

### Overview of the study area

The experimental site was in Xixiu District, Anshun City, Guizhou Province (106°5′59″ E, 26°6′29″ N, altitude 1271 m). The site has a subtropical humid monsoon climate, annual rainfall of 968–1309 mm, and a mean annual temperature of 14.5°C. The field experiment began in March 2021 and ended in October.The experimental site had yellow paddy soil, and single-season rice planting was performed. The soil properties were pH 5.19, organic matter content 35.16 g/kg, available nitrogen content 177.03 mg/kg, available phosphorus content 9.14 mg/kg, and available potassium content 96.09 mg/kg.

### Experimental design and test materials

(**Table 1**) Four treatments were used. These were no fertilization (CK), single chemical fertilization (NPK), organic fertilizer replacing 50% chemical fertilization (1/2NPKM), and only organic fertilization (M). Each treatment was performed in triplicate, and thus 12 plots were used. The plots were separated by cement ridges. Each plot had an area of 24 m^2^. The treatments were performed on randomly selected plots.

**Table 1:** Amounts of fertilizers used in the field experiment treatments.

treatments	fertilization dosage/(kg/hm^2^)			organic fertilizer/
	N	P2O5	K2O	(t/hm2)
CK	0	0	0	0
M	0	0	0	7.50
NPK	150	40	100	0
1/2NPKM	75	20	50	3.75

Rice seedlings were grown in April 2021 and transplanted in June 2021. The seedlings were planted 20 cm apart in rows 30 cm apart. Phosphate and organic fertilizers were applied once as base fertilizers. The base to nitrogen topdressing fertilizer ratio was 5:5. Topdressing was performed when the tillering:panicle ratio was 3:2. Topdressing was also performed at the heading stage, and other management practices were consistent with local paddy field management practices.

The fertilizers were urea (46.2% N), calcium superphosphate (16.5% P_2_O_5_), and potassium chloride (60.3% K_2_O). The organic fertilizer was a commercial organic fertilizer with an organic matter content of 45.1%, a N content of 2.02%, a P_2_O_5_ content of 2.2%, and a K_2_O content of 1.1%. The rice varieties are Jincheng Yahe (Jincheng 2A × Yahe), Tianxiangdao (first-class high-quality) national approved rice 20206073, Sichuan Tianyu Seed Industry Co., Ltd., one-season mid-season rice, and the whole growth period is 152 days.

### Soil sample collection

Soil samples were collected in mid- and late-September 2021. Five sampling points in each plot were selected at random, and a 0–20 cm deep soil sample was collected from each point using a soil drill. Each soil sample was divided into two parts. One part was passed through a 2 mm sieve and stored at 4 °C before being used to determine the MBC content and for organic carbon mineralization culture tests. The other part was dried in the dark at room temperature and passed through 2 and 0.25 mm sieves before the SOC and easily oxidized organic carbon contents were determined.

### Experimental site and permit requirements

The experiment was conducted in Xixiu District, Anshun City, Guizhou Province (106°5′59′′E, 26°6′29′′N), which has a subtropical monsoon humid climate. According to relevant Chinese laws and regulations, the experimental site is a general agricultural area and does not involve nature reserves, ecological redline areas, or other regions that require special permits. Therefore, no special field access permit is needed for this experiment. During the experiment, we followed local agricultural management practices and coordinated with the local agricultural department to ensure the smooth progress of the experiment. All experimental activities comply with local environmental protection and agricultural practice standards and will not have adverse impacts on the environment.

### Analytical methods

(1) Soil properties. The pH was determined using a composite electrode method, and the organic carbon content was determined using the K_2_Cr_2_O_7_–H_2_SO_4_ external heating method, and the total nitrogen content was determined using the Kjeldahl method, and the alkaline hydrolysable nitrogen content was determined using the alkaline hydrolysis diffusion method, and the available phosphorus content was determined using the NaHCO_3_ method, and the available potassium content was determined using the flame photometric method [24].
(2) Active carbon components of soil. The MBC content was determined using the chloroform fumigation method with extraction using 0.5 mol/L K_2_SO_4_ [25], and the DOC content was determined using a previously published method [26], and the ROC content was determined using the 0.333 mol/L KMnO_4_ oxidation method [27].
(3) SOC mineralization cultures [14]. The alkali absorption method was used. A 30.0 g aliquot of fresh soil was added to a 50 mL beaker and the water content was adjusted to 30% of the field capacity. The beaker was placed at the bottom of a 1000 mL culture flask. The soil was then kept in the dark at 25 °C for 7 d. A lye absorption cup containing 10 mL of 0.1 mol/L NaOH was then placed at the bottom of the flask. The flask was then sealed and kept in the dark at 25 °C. Each treatment was repeated three times. Six blank controls were analyzed. A total of 42 groups of mineralization cultures were performed. The absorption cup was removed and a fresh lye absorption cup was added on days6,12,18,24,30,36,42,48,54,60, and water was added to the soil to maintain the soil moisture content. A 2 mL aliquot of 1 mol/LBaCl_2_ was added to a used lye absorption cup, then two drops of phenolphthalein reagent were added. Then, 0.1 mol/L HCl (calibrated with borax) was used to titrate the mixture until the color disappeared. SOC mineralization was determined from the amount of CO_2_ released.
(4) Ethical statement: This study strictly abides by the ethical norms of academic research to ensure the legitimacy and morality of all research activities.

### Calculation and statistical analysis methods

#### Calculations.


$$C=C_{HCL}*\left(V_{0}-V_{1}\right)*22/0.03$$ (1)

$$C_{n}=\sum c$$ (2)

$$C_{v}=C_{n}/t$$ (3)

$$C_{m}=C_{n}/S_{SOC}$$ (4)

$$\;qMB=MBC/S_{SOC}$$ (5)

where *C* represents the amount of soil organic carbon mineralized (mg/kg); where *C (HCl)* is the hydrochloric acid concentration (mol/L), *V*_*0*_ is the blank titration volume (mL), and *V*_*1*_ is the volume of hydrochloric acid consumed (mL); *Cn* is the cumulative mineralization of soil organic carbon; *Cv* is the cumulative mineralization rate of soil organic carbon. (mg/(kg·d)); *t* is the incubation time(d); *Cm* is the cumulative mineralization rate of soil organic carbon (%); *S*_*SOC*_ is the soil organic carbon content (g/kg); *qMB* is soil microbial quotient (%); *MBC* is the soil microbial biomass carbon content (g/kg).

### Statistical analysis

Excel 2016 was used to perform statistical analyses of the data. Origin2022 software was used to create plots. One-way analyses of variance were performed using SPSS 22.0 software to assess differences between different treatments (p < 0.05).The results of the determination and analysis are detailed in S1 and S2 Tables in the support information.

## Results

### Effects of applying organic fertilizer instead of chemical fertilizer on soil properties

(**Table 2**) shows that the SOC, total nitrogen, available phosphorus, and available potassium contents increased after each treatment. The SOC contents were 7.84%, 11.64%, and 8.68% higher (p < 0.05) after the NPK, 1/2NPKM, and M treatments, respectively, than after the CK treatment. The total nitrogen contents were 9.05% and 3.88% higher and the available potassium contents were 4.62% and 16.38% lower (p < 0.05) after the NPK, 1/2NPKM, and M treatments, respectively, than after the CK treatment, The pH of 1/ 2NPKM treatment was significantly higher than that of the other three treatments.

**Table 2:** Effects of organic fertilizer replacing chemical fertilizer on soil organic carbon and nutrient contents.

treatments	SOC(g/kg)	pH	TN(g/kg)	AP(mg/kg)	AK(mg/kg)
CK	16.41 ± 0.24b	5.94 ± 0.20b	1.98 ± 0.13b	10.54 ± 0.14b	62.17 ± 3.01c
NPK	17.69 ± 0.09a	6.30 ± 0.06b	2.06 ± 0.10ab	13.54 ± 0.31a	86.50 ± 2.78a
1/2NPKM	18.32 ± 0.65a	6.56 ± 0.67a	2.30 ± 0.16a	13.11 ± 0.67a	82.50 ± 1.00a
M	17.83 ± 0.96a	6.24 ± 0.03b	2.14 ± 0.08ab	13.47 ± 0.38a	72.33 ± 1.76b

Note: Significant differences (p < 0.05) between values in a column for the different fertilization treatments are indicated by different lowercase letters.

### Effects of replacing chemical fertilizer with organic fertilizer on SOC mineralization

#### Cumulative SOC mineralization rate.

**Fig 1:**
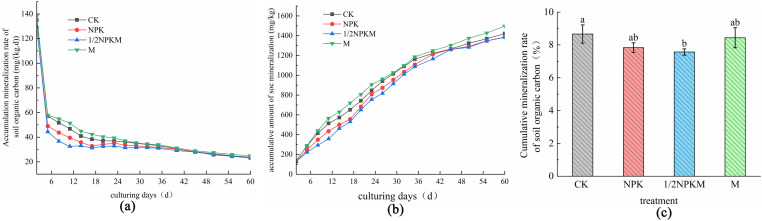
Characteristics of soil organic carbon mineralization. (a) cumulative soil organic carbon mineralization rate, (b) cumulative amount of soil organic carbon mineralization, and (c) Cumulative mineralization rate of soil organic carbon.

The cumulative SOC mineralization rate for each treatment decreased during the 60 d incubation period **(**Fig 1a). The cumulative SOC mineralization rate decreased strongly in the early stage (2–5 d), and had decreased at 5 d to 34.33%–42.33% of the rate at 2 d. In the middle stage (5–26 d), the cumulative SOC mineralization rate decreased slightly, and at 26 d was 24.31%–26.80% of the rate at 2 d. In the later stage (26–60 d), the cumulative SOC mineralization rate had become stable, and at 60 d was 17.55%–18.04% of the rate at 2 d. The cumulative SOC mineralization rates for the treatments decreased in the order M > CK > NPK > 1/2NPKM. The cumulative SOC mineralization rate (y) and incubation time (t) during the incubation period had the logarithmic relationship *y = b + k ln(t) (p < 0.05)* (Table 3).

**Table 3:** Regression equations for the cumulative soil organic carbon mineralization rates and the mineralization kinetic equation parameters.

treatments	regression equation	R^2^	C_0_/(g/kg)	K/ (d^-1^)	T_1/2_/ (d)	C_0_/SOC/ (%)	R^2^
CK	y = 73.58 -3.50ln(t)	0.481^**^	1.67	0.032	21.66	10.20	0.994^**^
NPK	y = 65.58 -2.97ln(t)	0.392^**^	1.83	0.025	27.73	10.35	0.992^**^
1/2NPKM	y = 61.20 -2.69ln(t)	0.311^**^	2.05	0.020	34.83	11.19	0.991^**^
M	y = 77.20 -2.70ln(t)	0.505^**^	1.70	0.033	21.00	9.52	0.996^**^

Notes: y = CO_2_ production rate (mg/ (kg·d)), x = days of culture (d), ** very significant (p < 0.01). (1) C_t_ = cumulative organic carbon mineralization, (2) C_0_ = amount of potentially mineralizable organic carbon, (3) k = organic carbon mineralization rate constant, (4) T_1/2_ = half-life, (5) potentially mineralizable organic carbon to total organic carbon ratio C_0_/ SOC.

### Cumulative amount of SOC mineralized

(Fig 1b**)** shows that the cumulative amount of SOC mineralization for each treatment increased as the incubation time increased. The cumulative amount of SOC mineralization for the treatments decreased in the order of M > CK > NPK > 1/2NPKM. The cumulative amount of SOC mineralized was 2.65% and 0.17% lower (p > 0.05) for the 1/2NPKM treatment than the CK and NPK treatments, respectively, at the end of the incubation period but 5.33% (p > 0.05) and 8.01% (p < 0.05) higher for the M treatment than the CK and NPK treatments, respectively.

(Table 3) shows that the cumulative amount of SOC mineralization (C) and culture time (t) were described well by the first-order kinetic equation C = C_0_ (1 − e^−kt^) (p < 0.01). The potentially mineralizable organic carbon content C_0_, mineralization rate constant *k*, half-life T_1/2_, and C_0_/SOC for the different treatments were different after 60 d of culturing. The mineralization rate constants *k* were 0.02–0.03 d^−^1, and the half-lives T_1/2_ were 21.00–34.83 d. The turnover rates for the treatments decreased in the order of M > CK > NPK > 1/2NPKM, and the half-lives increased in the order of M < CK < NPK < 1/2NPKM. C_0_, T_1/2_, and C_0_/SOC were highest and k was lowest for the 1/2NPKM treatment. C_0_ was 22.80%, 12.02%, and 20.59% higher and C_0_/SOC was 9.70%, 8.12%, and 17.45% higher for the 1/2NPKM treatment than the CK, NPK, and M treatments, respectively. This indicated that using 50% organic fertilizer and 50% chemical fertilizer increased the SOC turnover time, decreased the turnover rate, and inhibited SOC mineralization.

### Cumulative SOC mineralization rate

As shown in (**Fig 1c**), the cumulative mineralization rate was 12.65% lower (p < 0.05) for the 1/2NPKM and M treatments than the CK treatment. The cumulative mineralization rate was 3.40% lower (p > 0.05) for the 1/2NPKM treatment and 7.67% higher (p > 0.05) for the M treatment than the NPK treatment. This result indicated that the 1/2NPKM treatment decreased the SOC mineralization rate and increased the soil carbon sequestration capacity.

### Effects of adding organic fertilizer instead of chemical fertilizer on active SOC

#### Effect of adding organic fertilizer instead of chemical fertilizer on the proportion of active SOC.

The ROC/SOC and DOC/SOC ratios varied between the treatments in a similar way to the SOC content **(**Table 4). The ROC/SOC ratio was 11.46% and 9.32% higher (p < 0.05) for the 1/2NPKM and M treatments, respectively, than the CK treatment. The DOC/SOC ratio was 39.64% and 27.93% higher (p < 0.05) for the 1/2NPKM and M treatments, respectively, than the CK treatment. The qMB was 5.32% higher for the 1/2NPKM treatment than the CK treatment, and the qMB was 1.96% lower for the M treatment than the CK treatment. The ROC/SOC ratio was 2.24% and 4.24% lower for the 1/2NPKM and M treatments, respectively, than the NPK treatment. The DOC/SOC ratio was 5.16% higher for the 1/2NPKM treatment than the NPK treatment and 3.52% lower for the M treatment than the NPK treatment. The qMB was 6.23% lower (p > 0.05) and 12.72% lower (p < 0.05) for the 1/2NPKM and M treatments, respectively, than the NPK treatment.

**Table 4:** Soil organic carbon contents and proportions of active organic carbon components in the total organic carbon.

treatments	ROC/SOC (%)	DOC/SOC (%)	qMB (%)
CK	27.43 ± 1.33b	0.37 ± 0.03b	1.19 ± 0.04b
NPK	31.25 ± 1.88a	0.49 ± 0.02a	1.34 ± 0.06a
1/2NPKM	30.57 ± 0.66a	0.52 ± 0.01a	1.25 ± 0.06ab
M	29.98 ± 0.98a	0.47 ± 0.06a	1.17 ± 0.09b

### Effects of replacing chemical fertilizer with organic fertilizer on active soil organic carbon contents before and after mineralization

**Fig 2:**
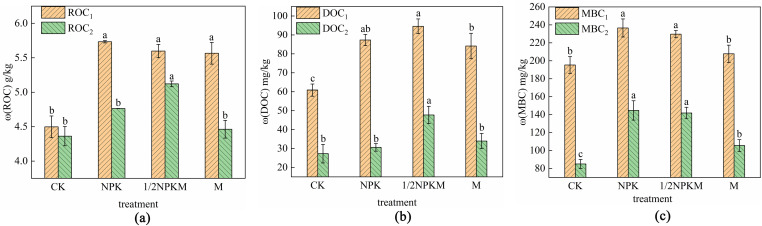
Active organic carbon components before and after mineralization. (a)The contents of readily oxidizable organic carbon (ROC), (b)dissolved organic carbon (DOC) and (c)microbial biomass carbon (MBC) were determined. Notes: 1 indicates before mineralization, 2 indicates after mineralization, different lowercase letters indicate significant differences between treatments (p < 0.05).

As shown in (**Fig 2a**), before mineralization, the ROC content was of 24.46% and 18.64% higher (p < 0.05) for the 1/2NPKM and M treatments, respectively, than the CK treatment. After mineralization, the ROC content was 19.34% and 2.34% higher for the 1/2NPKM and M treatments, respectively, than the CK treatment. The ROC content was 10.32% higher (p < 0.05) for the 1/2NPKM treatment than the NPK treatment. The ROC contents were 5.49%–18.63% lower after than before mineralization for each treatment.

As shown in (**Fig 2b**), before mineralization, the DOC content was 55.45% and 8.31% higher (p < 0.05) for the 1/2NPKM treatment than the CK and NPK treatments, respectively. After mineralization, the DOC content was 74.98% and 56.03% higher (p < 0.05) for the 1/2NPKM treatment than the CK and NPK treatments, respectively. The DOC contents for the treatments were 49.56%–64.99% lower after than before mineralization.

As shown in **(Fig 2c)**, before mineralization, the MBC content was 17.60% and 6.40% higher (p > 0.05) for the 1/2NPKM and M treatments, respectively, than the CK treatment. The MBC content was 2.90% and 12.15% (p < 0.05) lower for the 1/2NPKM and M treatments, respectively, than the NPK treatment. After mineralization, the MBC content was 66.83% and 24.16% higher (p < 0.05) for the 1/2NPKM and M treatments, respectively, than the CK treatment. The MBC content was 1.98% and 27.05% lower (p < 0.05) for the 1/2NPKM and M treatments, respectively, than the NPK treatment. The MBC contents for the treatments were 38.26%–56.48% lower after than before mineralization.

### Relationships between SOC, active organic carbon fractions, and organic carbon mineralization

**Fig 3:**
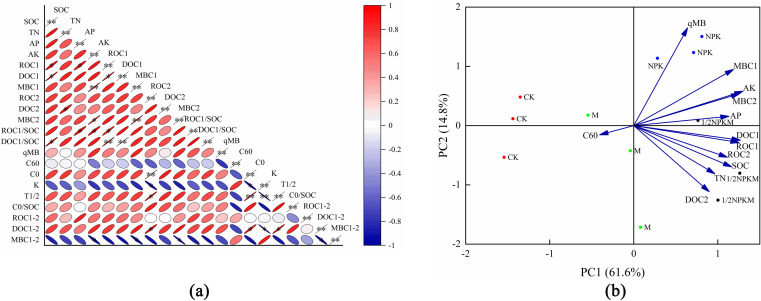
Correlation and principal component analysis. (a) Correlation analysis plot,(b) Principal component analysis plot. Notes: In plot (a), the larger the ellipse the lower the correlation coefficient and the smaller the ellipse the higher the correlation coefficient. Red indicates a positive correlation, blue indicates a negative correlation, the deeper the color the stronger the correlation, and the paler the color the weaker the correlation. * indicates p < 0.05, ** indicates p < 0.01. C60 (%) is the cumulative soil organic carbon mineralization rate at 60 d.

The correlation analysis results (Fig 3a) indicated that the cumulative SOC mineralization rate significantly negatively correlated with the MBC_1_ content (p < 0.01) and significantly negatively correlated with the MBC_2_ content (p < 0.05) but did not significantly correlate with the other parameters (p > 0.05). The ROC_2_ content significantly positively correlated with C_0_ (p < 0.01), significantly negatively correlated with k (p < 0.05), and significantly positively correlated with T_1/2_ (p < 0.05). This indicated that the SOC cumulative mineralization rate was closely related to changes that occurred during MBC mineralization but had a weak relationship with changes in the other parameters. The ROC_2_ content was closely related to C_0_, k, and T_1/2_ for the SOC mineralization kinetic equation.

The principal component dimension reduction method was used to investigate the contributions of the different fertilization treatments to differences in the SOC, pH,total nitrogen, available phosphorus, available potassium, DOC_1_, DOC_2_, ROC_1_, ROC_2_, MBC_1_, and MBC_2_ contents and qMB and C_60_. The results (Fig 3b) indicated that principal components PC1 and PC2 jointly explained 75.6% of the variations, with PC1 explaining 61.9% and PC2 explaining 13.7% of the variations. The NPK, 1/2NPKM, and M treatment datapoints mainly had positive PC1 values but the CK treatment datapoints mainly had negative PC1 values, indicating that the SOC, total nitrogen, available phosphorus, DOC, ROC, and MBC contents were more strongly affected by the NPK, 1/2NPKM, and M treatments than the CK treatment. The NPK and 1/2NPKM treatments increased the SOC and active carbon component contents overall. The SOC and active carbon component contents were affected more by the 1/2NPKM treatment than the NPK treatment. The M treatment increased cumulative SOC mineralization but the 1/2NPKM treatment decreased cumulative SOC mineralization, indicating that the M treatment promoted but the 1/2NPKM treatment inhibited SOC mineralization.

## Discussion

### Effects of applying organic fertilizer instead of chemical fertilizer on soil properties

The results indicated that the SOC, total nitrogen, available phosphorus, and available potassium contents were higher after fertilization than without fertilization, which agreed with the results of a previous study [28]. This may have been because applying organic fertilizer caused mineralization to occur through the activities of microorganisms and that these activities caused mineral nutrients to be released. Urea applied to soil is converted by urease into ammonium and nitrate. Calcium superphosphate, potassium chloride, and organic fertilizer add large amounts of organic matter, nitrogen, phosphorus, and potassium to soil, and thus increase the nutrient contents of the soil [29].

In our study, the SOC and total nitrogen contents were higher for the 1/2NPKM treatment than the other three treatments and the available phosphorus and available potassium contents were lower for the 1/2NPKM treatment than the NPK treatment, consistent with the results of a previous study [30]. This may have been because the organic fertilizer contained large amounts of organic matter and certain nutrients. Organic fertilizer applied to soil is decomposed by soil microorganisms, which increases the SOC and soil total nitrogen contents. Organic fertilizer continually provides phosphorus and potassium, which are required for plant growth, whereas chemical fertilizers tend to be applied in single applications, meaning more infiltration into the soil will occur.

### Effects of replacing chemical fertilizer with organic fertilizer on SOC mineralization

Fertilization is a key agricultural management practice that can affect SOC mineralization processes [31]. Our results indicated that the cumulative SOC mineralization rate for each treatment could be divided into three stages, rapid mineralization in the early stage, a slow decrease in the rate in the middle stage, and stabilization in the late stage, consistent with the results of a previous study [32]. This may be because soil contains readily decomposed organic carbon sources that are mineralized in the early stage. The number of microorganisms and microorganism activity will therefore increase in the early stage, and the cumulative SOC mineralization rate will increase. In the middle stage, the amount of readily decomposed organic carbon compounds would have decreased, and the relative proportion of complex organic carbon compounds that are difficult to decompose would have increased, and thus microorganism activity would have decreased and the cumulative mineralization rate decreased. In the late stage, the microorganism number and activity would have been low, and the cumulative SOC mineralization rate stabilized [33].

The cumulative mineralization of soil organic carbon (SOC) was higher in the organic fertilizer-only treatment (M treatment) than in the other treatments, which is consistent with the findings of Liu et al [34]. In the rhizosphere soil of maize, the cumulative mineralization of soil organic carbon (SOC) in the treatment with single application of cattle manure was significantly higher than that in other treatments [35]. This may have been because the test soil was acidic, which would not have been conducive to soil microorganism activity. Applying organic fertilizer would effectively have increased the soil pH and made the soil environment more suitable for microbe growth, and thus would have accelerated organic matter decomposition and CO_2_ release by microorganisms [36]. Secondly, the application of chemical fertilizers indirectly supplies nutrients to microorganisms, enriching the composition of soil microbial communities. The presence of microbial communities that are not conducive to soil organic carbon mineralization may lead to a reduction in the cumulative mineralization of soil organic carbon (SOC) [37]. Manure and organic fertilizer contain more unstable organic matter. After being input into soil, the humification index of soluble organic matter of organic manure is lower than that of soil soluble organic matter [38]. In particular, the application of organic fertilizer increased the content of soluble organic matter and reduced the complexity of its structure, thereby promoting the mineralization of soil organic carbon [39].The M treatment markedly increased the amount of CO_2_ released and the cumulative SOC mineralization rate, which would not have been conducive to carbon sequestration and would have increased greenhouse gas emissions. A reasonable decrease in the proportion of organic fertilizer used to replace chemical fertilizer would be beneficial to SOC accumulation in farmland soil. From the perspective of carbon sequestration and decreasing greenhouse gas emissions, the 1/2NPKM treatment was ideal. After the organic fertilizer is applied to the soil, the microbial decomposition of organic matter requires appropriate water, temperature, oxygen, etc. The inorganic anions in the 1/2NPKM treated chemical fertilizer and the organic anions in the organic fertilizer promote the desorption of organic carbon in the soil itself. Under the condition of long-term flooding, the foreign carbon source and crop stubble residues are not completely decomposed by microorganisms, but only increase the content of active carbon components, thus reducing the mineralization of organic carbon [14,40].

### Effects of replacing chemical fertilizer with organic fertilizer on the active organic carbon fractions

Many studies have found that applying organic fertilizer can increase the active organic carbon component contents of soil, and our results also confirmed this. This is because organic fertilizers contain a higher amount of labile organic matter, which promotes the transformation of inert plant-derived carbon into more decomposable forms of organic carbon, thereby expanding the pool of active organic carbon in the soil [41]. The active carbon pools in manure and organic fertilizers usually have smaller molecular weights and simpler components, which are more easily accessible and utilized by microorganisms, and the organic matter in the active organic components is more easily degraded, thereby increasing the content of active carbon components [42,21]. The DOC content of soil was increased more by the 1/2NPKM treatment than the other treatments and the ROC and MBC contents were increased more by the NPK treatment than the other treatments, consistent with the results of previous studies [43,44]. This may be because DOC is a key source of carbon for maintaining microbial metabolism [10]. Organic fertilizer can replace some chemical fertilizer in promoting the transformation of stable organic carbon into active organic carbon and will therefore increase the active organic carbon component contents of soil [45]. A single application of chemical fertilizer can provide nutrients to soil microorganisms, increase the microbial community abundance and enzyme activity, increase plant stubble degradation and root exudate production, and indirectly increase the ROC and MBC contents [46]. After the culture period, the active organic carbon component contents of the soil had decreased, and the ROC content had decreased the most, consistent with the results of a previous study [47]. This would have been because active carbon components were consumed as available carbon sources during SOC mineralization. ROC has a faster turnover rate and is more readily decomposed and used by microorganisms than other components [40].

### Relationships between active organic carbon fractions and organic carbon mineralization

Active organic carbon components in soil are effective carbon sources for microorganisms that cause organic carbon mineralization, and thus can strongly affect organic carbon mineralization [48]. We found that the cumulative SOC mineralization rate significantly negatively correlated with the MBC content and the ROC content significantly positively correlated with C_0_, significantly negatively correlated with k, and significantly positively correlated with T_1/2_, consistent with the results of a previous study [49].

The cumulative mineralization rate weakly correlated with the SOC, ROC, and DOC contents, possibly because of variations in the soil utilization types and soil structures, which would have caused variations in the microenvironments for soil microorganisms. The microbe community structures in different microenvironments will be different. Microorganisms using MBC were the most active, and microorganisms using ROC and DOC were least active [50], indicating that SOC mineralization was closely related to the active organic carbon component contents. The cumulative SOC mineralization rate did not significantly correlate with the ROC and DOC contents but did significantly negatively correlate with the initial MBC change value. This would have been because ROC and DOC are not strongly involved in the organic carbon mineralization process even though they are effective carbon sources for microorganisms. MBC, a direct carbon source for microorganisms, is used more than ROC and DOC in the SOC mineralization process [51]. Increasing the MBC content of farmland soil is therefore an important way of improving soil quality. The SOC mineralization process is affected by many factors. Soil aggregates, humus, and soil sediments can also affect the SOC mineralization process.

## Conclusions

Replacing 50% chemical fertilizer with organic fertilizer significantly increased the content of soil organic carbon and active carbon components, slowed down the mineralization of soil organic carbon, and increased the stability of soil organic carbon. Soil organic carbon mineralization was negatively correlated with SOC and active carbon fractions, and SOC was positively correlated with active carbon fractions. The cumulative mineralization rate of soil organic carbon was closely related to MBC, and ROC and MBC were the main carbon sources for soil organic carbon mineralization. In the future, we should further study the in-situ organic carbon mineralization in the field, measure the release of organic carbon mineralization, and analyze the effect of fertilization on organic carbon mineralization and active carbon components, so as to find the best field fertilization method.

## Supporting information

S1 Table Characteristics of soil organic carbon mineralization for 45 days. (XLSX)

S2 Table Soil active organic carbon and basic nutrient content under different fertilization treatments. (XLSX)
